# Dynamic methods for ongoing assessment of site-level risk in risk-based monitoring of clinical trials: A scoping review

**DOI:** 10.1177/1740774520976561

**Published:** 2021-02-20

**Authors:** William J Cragg, Caroline Hurley, Victoria Yorke-Edwards, Sally P Stenning

**Affiliations:** 1 MRC Clinical Trials Unit at UCL, London, UK; 2 Clinical Trials Research Unit, Leeds Institute of Clinical Trials Research, University of Leeds, Leeds, UK; 3 Health Research Board-Trials Methodology Research Network (HRB-TMRN), National University of Ireland Galway, Galway, Ireland

**Keywords:** Trial monitoring, risk-based monitoring, triggered monitoring, central statistical monitoring, Good Clinical Practice, research misconduct, data fabrication

## Abstract

**Background/Aims:**

It is increasingly recognised that reliance on frequent site visits for monitoring clinical trials is inefficient. Regulators and trialists have recently encouraged more risk-based monitoring. Risk assessment should take place before a trial begins to define the overarching monitoring strategy. It can also be done on an ongoing basis, to target sites for monitoring activity. Various methods have been proposed for such prioritisation, often using terms like ‘central statistical monitoring’, ‘triggered monitoring’ or, as in the International Conference on Harmonization Good Clinical Practice guidance, ‘targeted on-site monitoring’. We conducted a scoping review to identify such methods, to establish if any were supported by adequate evidence to allow wider implementation, and to guide future developments in this field of research.

**Methods:**

We used seven publication databases, two sets of methodological conference abstracts and an Internet search engine to identify methods for using centrally held trial data to assess site conduct during a trial. We included only reports in English, and excluded reports published before 1996 or not directly relevant to our research question. We used reference and citation searches to find additional relevant reports. We extracted data using a predefined template. We contacted authors to request additional information about included reports.

**Results:**

We included 30 reports in our final dataset, of which 21 were peer-reviewed publications. In all, 20 reports described central statistical monitoring methods (of which 7 focussed on detection of fraud or misconduct) and 9 described triggered monitoring methods; 21 reports included some assessment of their methods’ effectiveness, typically exploring the methods’ characteristics using real trial data without known integrity issues. Of the 21 with some effectiveness assessment, most contained limited information about whether or not concerns identified through central monitoring constituted meaningful problems. Several reports demonstrated good classification ability based on more than one classification statistic, but never without caveats of unclear reporting or other classification statistics being low or unavailable. Some reports commented on cost savings from reduced on-site monitoring, but none gave detailed costings for the development and maintenance of central monitoring methods themselves.

**Conclusion:**

Our review identified various proposed methods, some of which could be combined within the same trial. The apparent emphasis on fraud detection may not be proportionate in all trial settings. Despite some promising evidence and some self-justifying benefits for data cleaning activity, many proposed methods have limitations that may currently prevent their routine use for targeting trial monitoring activity. The implementation costs, or uncertainty about these, may also be a barrier. We make recommendations for how the evidence-base supporting these methods could be improved.

## Introduction

Monitoring, a major component of assuring the quality of clinical trials, has traditionally relied on frequent on-site monitoring visits,^1^ particularly to facilitate sometimes extensive source data verification (SDV).^2^ However, it is increasingly recognised that this model may be inefficient and unnecessary in many cases,^3,4^ with trialists questioning the value of 100% SDV.^5–7^ In recent years, regulators^8–10^ and trialists^1,11^ have proposed a risk-based approach to monitoring, whereby monitoring methods, including the frequency and nature of on-site visits, vary across trials depending on the risks specific to each one. The support of regulators is encouraging, indicating that risk-based methods might be implemented even in clinical trials of investigational medicinal products, that is, those historically subject to particular regulatory control and claimed to suffer under a ‘regulatory burden’.^12,13^

Risk-based monitoring methods can be applied at different stages of a trial. Pre-trial risk assessments can help define the overarching strategies appropriate to the trial’s risks. In some models,^14,15^ this is predominantly a one-off assessment during trial set-up. However, it is also possible to modify the monitoring strategy, or incorporate flexibility, based on emerging risks during the course of the trial.^16^

Risk-based monitoring is often associated with fewer on-site visits than ‘traditional’ monitoring.^17^ Although effective central monitoring methods alone could, in some respects, provide adequate trial monitoring in place of visits, on-site visits offer particular benefits over central monitoring. These include, for example, the ability to access site-held source data (such as patients’ medical notes, although some have suggested these might be accessed remotely instead),^18–20^ conduct in-person facility review^21^ or assess processes such as informed consent.^22^ On-site visits may also be necessary to investigate potential fraudulent activity. In a risk-based monitoring framework, visits to sites may not be routine, but can be based on assessed risk; we therefore need methods to assess site-level risk on an ongoing basis. We can interpret these methods as assessing the risk of *not going to site now.* If the risk seems too high, a visit – or some other corrective action – is triggered. Methods of this kind have been referred to using various terms, including ‘triggered monitoring’^16^ or, as in ICH Good Clinical Practice guidance, ‘targeted monitoring’,^23^ and may employ data-driven approaches from methods known collectively as ‘central statistical monitoring’,^24^ or more subjective assessments.^16,25,26^

A recent systematic review has established the breadth of tools available to assess overall trial risk (and to use this assessment to define the monitoring strategy) in the set-up stage,^27^ but so far there has been no such exercise for methods to assess ongoing site-level risk once a trial has started. We conducted a scoping review^28^ to identify and characterise available methods.

Our aims were (a) for trialists, to establish if any published methods were supported by adequate evidence to support implementation in routine practice and (b) for researchers in this area, to consolidate the existing evidence and point towards future developments in this growing field.

## Methods

We conducted a scoping review to identify methods for using centrally held clinical trial data to assess site-level risk of deviations from Good Clinical Practice or the trial protocol, or research misconduct, and thereby to target sites for further monitoring activity. We chose scoping review methodology as we anticipated finding a variety of results, and we wanted to characterise the extent, range and nature of research activity.^29^ There is no published protocol for this scoping review.

### Eligibility criteria

We defined our eligibility criteria before beginning any searches, with minor refinements (mainly to the exclusion criteria) after search strategy piloting.

We included original reports (a) describing methods for using centrally held data (i.e. at the trial coordinating centre) to assess, in ongoing trials, site-level risk of protocol or Good Clinical Practice deviation, risk of data fabrication or research misconduct, or to target sites in some other way for corrective action based on assessed risk (regardless of whether the corrective action involved an on-site monitoring visit or not); (b) with methods described in enough detail that we considered them – subjectively – reproducible; (c) either published in peer-reviewed journals or available as grey literature; (d) about clinical trials, not limited to trials of Investigational Medicinal Products; and (e) in English.

We excluded reports (a) published before 1996 (the year of the first version of the International Conference on Harmonization Good Clinical Practice Guidance, E6[R1])^30^; (b) about quality assurance only in the context of intervention fidelity^31^ or ‘rater differences’^32^ for subjective trial outcome measures; (c) about ‘data monitoring’ in general, for example, data monitoring committees, or ‘monitoring’ in any sense other than the Good Clinical Practice sense, for example, clinical monitoring; (d) focusing only on trial recruitment; (e) about more efficient alternatives to standard on-site activity, for example, remote SDV; and (f) about site selection during trial set-up.

### Information sources and search strategies

#### Database searches

We designed search strategies for the following databases: (a) PubMed, (b) Embase (Ovid), (c) Medline (Ovid), (d) Web of Science (Clarivate Analytics), (e) CINAHL, (f) Cochrane Central and (g) Scopus.

Full database searches took place on 23 October 2017 (run and extracted by W.J.C.). The search strategy for Medline is given in the Supplementary Information. We developed our search strategy following review of systematic reviews in this area^1,33^ to identify relevant search terms. The final search term combined searches around two concepts: clinical trials (using terms based on those used in a previous systematic review of monitoring methods)^33^ and targeted or risk-based clinical trial monitoring. No database filters were applied.

Both reviewers (W.C. and C.H.) imported results into reference management software and used in-built tools to remove duplicate entries. Both reviewers carried out initial title and abstract screening, producing an initial shortlist of potential papers. We reviewed and discussed these, using full-text reports where possible, to agree on a final list of relevant reports. Throughout the process, S.P.S. acted as third reviewer where required.

In order to ensure that our results were current, this element of the search strategy was repeated on 28 August 2018. W.J.C. ran the searches and conducted the title and abstract screening. A shortlist of potentially relevant reports was shared with S.P.S. and CH; S.P.S. and W.J.C. agreed on a final list of additional relevant reports from this repeated search.

#### Conference abstracts

We hand-searched for relevant conference abstracts from the first four International Clinical Trials Methodology Conferences (occurring between 2011 and 2017) and all annual meetings of the Society for Clinical Trials since 1996 (initial searches completed on 8 December 2017). Keywords used for the conference abstract, based on the key database search strategy terms, were ‘monitor’, ‘supervision’, ‘oversight’, ‘risk’, ‘performance’, ‘metric’, ‘quality’, ‘fraud’, ‘fabrication’ and ‘error’.

Both W.J.C. and C.H. performed the abstract searches. This produced an initial shortlist of potentially relevant abstracts. A final list was agreed upon through discussion, with S.P.S. acting as third reviewer where required.

#### Internet searches

We conducted structured searches through Google Internet search engine (searches carried out during 15–19 December 2017).

Google searches were performed without limitations or use of quotes. Search terms were based on the main database search: ‘Risk based monitoring’, ‘Risk adapted monitoring’, ‘Central monitoring’, ‘Central statistical monitoring’, ‘Triggered monitoring’, ‘Targeted monitoring’, ‘Performance metric’, ‘Site metric’, ‘Key risk indicator’, ‘Site performance’, ‘Centre performance’, ‘Detect fraud’ and ‘Detect fabrication’. We reviewed the results on the first 20 pages, or fewer if there were no relevant results on any three consecutive pages before this.

W.J.C. and C.H. conducted the searches. Any potential additions to the included list of reports were discussed and agreed upon, with S.P.S. acting as third reviewer where required.

#### References, citations and author contact

To identify other relevant reports, we reviewed references (manually) and citations (using Web of Science) of all papers included or considered for inclusion in the final results, and of review articles relevant to the topic. Whenever required, we contacted report authors to help ascertain if given reports should be included, and to ask about the availability of full-text articles.

### Data collection

We extracted data from full journal articles, where available. We recorded data into an Excel-based tool. W.J.C. carried out the final data collection used for this report, with S.P.S. double-checking all data for inclusion; consensus was reached on any areas of disagreement. Article authors were contacted (two attempts maximum) for missing descriptive data and further clarifications.

Our data collection template was designed and agreed prior to any data collection, with minor refinement after a first review of all relevant papers (a list of data collection variables is available as Supplementary Information). We collected descriptive data about each of the included reports, including any information on cost implications of the proposed methods.

When designing this study, although we predicted we would find a range of methods, we agreed that most of them would in essence address a classification problem, that is, methods to assign sites a status as ‘concerning’ or ‘not-concerning’, with a ‘true’ deviation status – that is, confirmed existence of meaningful problems – that could be uncovered by further review. The ‘gold standard’ reference test required to assess true status might be study-specific, but could be on-site monitoring or, if the true status was created through simulation, prior knowledge.

We considered a key measure of the reported methods’ effectiveness to be a demonstrated ability, ideally in a real-life setting, not only to detect ‘true’ sites of concern, but also to show with confidence that sites apparently not of concern are performing well. We therefore aimed to summarise the available information on classification, that is, any or all of specificity, sensitivity, positive and negative predictive value. We gathered the best reported classification statistics for each method, or, if this was not reported, used available statistics to calculate these. These calculations were verified by an independent statistician at the Medical Research Council Clinical Trials Unit at University College London.

We did not formally assess the quality of the studies. However, review of the QUADAS-2 tool for quality assessment in diagnostic accuracy studies^34^ informed development of our data collection template.

### Synthesis of results

The results are summarised descriptively rather than combined, as it was clear through preliminary review of the relevant papers that we would have a variety of study types.

## Results

**Figure 1.**
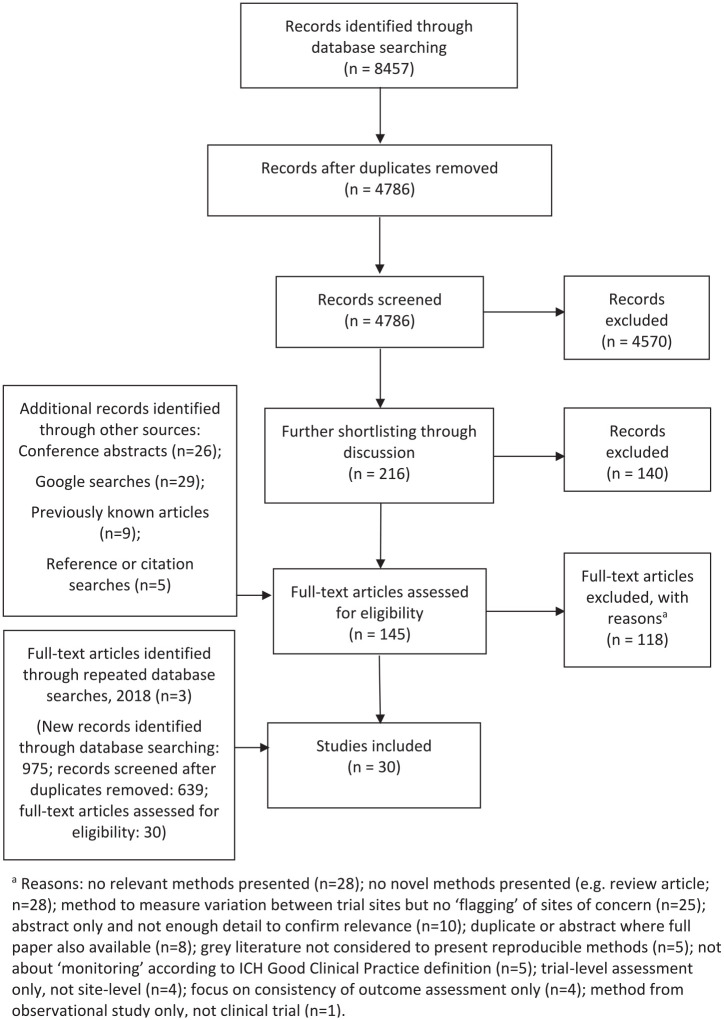
PRISMA flow diagram. ^a^Reasons: no relevant methods presented (n = 28); no novel methods presented (e.g. review article; n = 28); method to measure variation between trial sites but no ‘flagging’ of sites of concern (n = 25); abstract only and not enough detail to confirm relevance (n = 10); duplicate or abstract where full paper also available (n = 8); grey literature not considered to present reproducible methods (n = 5); not about ‘monitoring’ according to ICH Good Clinical Practice definition (n = 5); trial-level assessment only, not site-level (n = 4); focus on consistency of outcome assessment only (n = 4); method from observational study only, not clinical trial (n = 1).

**Figure 2.**
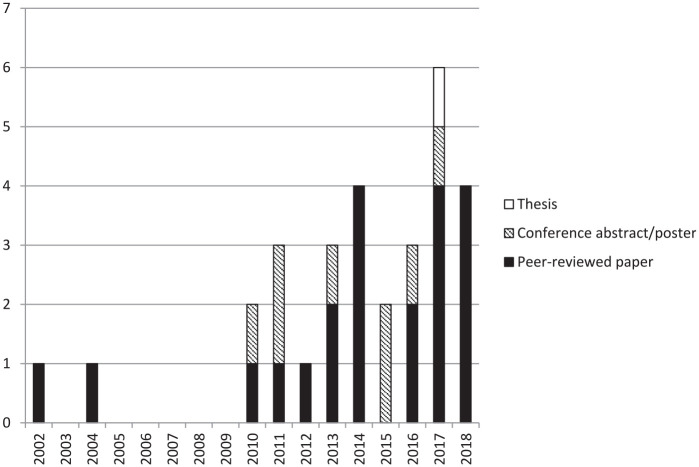
Publications by year and type.

Figure 1 gives a PRISMA flow diagram^35^ showing the different stages of the review. From the various data sources, we ultimately included 30 reports in our final dataset. Twenty-one of these are peer-reviewed publications. The results are characterised in Table 1 and listed in full in Table 2. Figure 2 shows reports by year of publication.

**Table 1.** General characteristics of included studies.

Characteristic	N (total = 30)	%
Publication year		
1996–2000	0	0
2000–2005	2	7
2006–2010	2	7
2010–2015	13	43
2016–2018	13	43
Type of source		
Peer-reviewed paper	21	70
Conference abstract or poster	8	27
Thesis	1	3
Disease setting of trial involved		
Cardiovascular disease	4	13
Emergency medicine	1	3
Haematology	1	3
Infectious diseases	1	3
Mental health	3	10
Neurology	1	3
Oncology	3	10
Ophthalmology	1	3
Renal disease	1	3
Respiratory disease	1	3
Unknown or no specific trial involved	13	43
Geographical setting of trials involved		
Brazil	1	3
International	7	23
Japan	1	3
North America	4	13
UK	2	7
Unknown or no specific trial involved	15	50
Use of Investigational Medicinal Product (IMP) in involved trials		
Involves IMP	14	47
No IMP	1	3
Unknown or no specific trial involved	15	50
Phase of trials involved		
Phase I	0	0
Phase II	1	3
Phases II and III	1	3
Phase III	9	30
Unknown or no specific trial involved	19	63
Status of investigational medicinal product used^a^		
Unlicensed	0	0
Licensed, used outside of its licensed indication	5	17
Licensed, used within its licensed indication	4	13
Unknown or no specific trial involved	22	73
Focus of work^a^		
Central statistical monitoring, focus on fraud or misconduct	7	23
Central statistical monitoring, general	13	43
Triggered monitoring	9	30
Other method(s) for highlighting sites at risk	2	7
Scope of work		
Description or development of method	9	30
Some assessment of methods’ effectiveness	21	70

a Categories not mutually exclusive.

**Table 2.** Full listing of all included reports.

Author(s)	Type of source	Focus of work	Scope of work
Agrafiotis et al.^36^	Peer-reviewed paper	Triggered monitoring	Some assessment of methods’ effectiveness
Almukhtar and Glassman^37^	Conference abstract/poster	Central statistical monitoring, general	Description or development of method
Atanu et al.^38^	Peer-reviewed paper	Central statistical monitoring, general	Description or development of method
Bailey et al.^39^	Conference abstract/poster	Triggered monitoring	Description or development of method
Bengtsson^40^	Thesis	Central statistical monitoring, general	Some assessment of methods’ effectiveness
Biglan et al.^41^	Conference abstract/poster	Triggered monitoring	Some assessment of methods’ effectiveness
Desmet et al.^42^	Peer-reviewed paper	Central statistical monitoring, general	Some assessment of methods’ effectiveness
Desmet et al.^43^	Peer-reviewed paper	Central statistical monitoring, general	Some assessment of methods’ effectiveness
Diani et al.^44^	Peer-reviewed paper	Triggered monitoring	Some assessment of methods’ effectiveness
Djali et al.^45^	Peer-reviewed paper	Other method(s) for highlighting sites at risk (combines site metric scores directly to flag sites of concern)	Some assessment of methods’ effectiveness
Dress et al.^46^	Conference abstract/poster	Triggered monitoring	Description or development of method
Edwards et al.^47^	Peer-reviewed paper	Central statistical monitoring with triggered monitoring	Some assessment of methods’ effectiveness
Kirkwood et al.^24^	Peer-reviewed paper	Central statistical monitoring, general	Some assessment of methods’ effectiveness
Knepper et al.^26^	Peer-reviewed paper	Central statistical monitoring, focus on fraud or misconduct	Some assessment of methods’ effectiveness
Knott et al.^48^	Conference abstract/poster	Central statistical monitoring, general	Some assessment of methods’ effectiveness
Kodama et al.^49^	Conference abstract/poster	Central statistical monitoring, focus on fraud or misconduct	Some assessment of methods’ effectiveness
Lindblad et al.^50^	Peer-reviewed paper	Central statistical monitoring, general	Some assessment of methods’ effectiveness
O’Kelly^51^	Peer-reviewed paper	Central statistical monitoring, focus on fraud or misconduct	Some assessment of methods’ effectiveness
Pogue et al.^52^	Peer-reviewed paper	Central statistical monitoring, focus on fraud or misconduct	Some assessment of methods’ effectiveness
Smith and Seltzer^53^	Peer-reviewed paper	Other method(s) for highlighting sites at risk (use of “statistical process control methodology” to combine per-site risk indicator scores)	Description or development of method
Stenning et al.^16^	Peer-reviewed paper	Triggered monitoring	Some assessment of methods’ effectiveness
Taylor et al.^54^	Peer-reviewed paper	Central statistical monitoring, focus on fraud or misconduct	Some assessment of methods’ effectiveness
Timmermans et al.^55^	Peer-reviewed paper	Central statistical monitoring, general	Some assessment of methods’ effectiveness
Tudur Smith et al.^25^	Peer-reviewed paper	Triggered monitoring	Description or development of method
Valdes-Marquez et al.^56^	Conference abstract/poster	Central statistical monitoring, general	Description or development of method
Valdes-Marquez et al.^57^	Conference abstract/poster	Central statistical monitoring, general	Description or development of method
Van den Bor et al.^58^	Peer-reviewed paper	Central statistical monitoring, focus on fraud or misconduct	Some assessment of methods’ effectiveness
Whitham, 2018^59^	Peer-reviewed paper	Triggered monitoring	Description or development of method
Wu and Carlsson^60^	Peer-reviewed paper	Central statistical monitoring, focus on fraud or misconduct	Some assessment of methods’ effectiveness
Zink et al.^61^	Peer-reviewed paper	Central statistical monitoring, general	Some assessment of methods’ effectiveness

Where information on trial intervention was available, methods had most often been used in Phase III trials of investigational medicinal products. The investigational medicinal product risk category,^62^ when known, was either ‘licensed and used within its licensed indication’, or ‘licensed and used outside its licensed indication’ (i.e. we found no reports involving trials of unlicensed investigational medicinal products).

We classified 20/30 of our results as central statistical monitoring methods, of which 7 focussed on detection of investigator fraud or research misconduct. We classified 9, including 1 of the 20 that used central statistical monitoring, as ‘triggered monitoring’, that is, review of each trial site against pre-set thresholds in key performance metrics, usually without any statistical testing. A final two did not fit into either of these categories; these involved using measured site metrics to directly compare sites against one another.^53,45^

A total of 21/30 reports included some assessment of the effectiveness of the methods; these are summarised in Table 3. The most common experimental designs were to explore the methods’ characteristics using real trial data with no known integrity issues (n = 9), and simulating data integrity problems at sites within real trial datasets and then using the method to try to identify the problem sites (n = 6).

**Table 3.** Types of assessments and evidence presented by reports that included some assessments of their methods’ effectiveness.

Author(s)	Case studies	Illustration of method(s) on data with no known issues	Assessment of methods’ ability to identify simulated problem sites	Assessment of methods’ ability to identify known problems in real trial data	Methods used in ongoing trial, results of on-site monitoring reported	Methods used in ongoing trial, effects reported on trial in general (e.g. in terms of cost or data quality)	Prospectively designed, controlled study to assess methods’ ability to target on-site monitoring visits to most problematic sites
Agrafiotis et al.^36^					X	X	
Bengtsson^40^		X					
Biglan et al.^41^					X	X	
Desmet et al.^42^	X	X	X				
Desmet et al.^43^		X	X				
Diani et al.^44^						X	
Djali et al.^45^	X						
Edwards et al.^47^	X						
Kirkwood et al.^24^		X	X				
Knepper et al.^26^			X				
Knott et al.^48^					X		
Kodama et al.^49^		X					
Lindblad et al.^50^				X			
O’Kelly^51^			X				
Pogue et al.^52^		X		X			
Stenning et al.^16^							X
Taylor et al.^54^		X					
Timmermans et al.^55^		X					
Van den Bor et al.^58^				X			
Wu and Carlsson^60^			X	X			
Zink et al.^61^		X					
Total	3	9	6	4	3	3	1

Of the 21 reports, 9 had no information about sites’‘true’ status, that is, whether the problems identified through central monitoring constitute meaningful problems (either recorded through on-site monitoring or audit activity, or known because statuses were created through simulation). One report^47^ only contained case studies, that is, partial and selective reporting. Seven^16,41,48,50,51,58,60^ had partial information, for example, some of sites’ true statuses were reported, but not all. Two explored classification ability through extensive simulation,^42,43^ and two had detailed information from a limited set of scenarios on the number of true and false positives and negatives.^26,52^

The best reported or deducible classification ability for the 11 papers with at least some information on sites’‘true’ status (excluding the case study paper) is shown in Table 4. Seven of these reports ascertained the ‘true’ status through on-site monitoring, audit or regulatory inspection and in three the ‘true’ status was known because it had been simulated. The final report^42^ presented both real and simulated scenarios. ‘Best’ classification statistics were reported or deducible in 8 of these reports (of the remaining 3, 1 did not report enough data to allow any calculations, and 2 reported extensive simulations that precluded reporting of a ‘best’ result).

**Table 4.** Best reported information on methods’ classification ability, where available or deducible.

Author(s)	Available information on methods’ classification abilities	Definition of ‘positive’ centres	‘True’ test status: real or simulated?	Test for ‘true’ centre status	Sensitivity^a^	Specificity^b^	Positive predictive value^c^	Negative predictive value^d^
Biglan et al.^41^	Partial (‘true’ status known for only one centre; total number of centres not known)	Not clearly defined^e^	Real	On-site monitoring	Unavailable due to limited data; report states that one ‘low-risk’ centre was visited and considered to be misclassified (i.e. should have been ‘medium risk’ or ‘high risk’). However, the total number of sites classified and visited (overall and within each risk category) is not known			
Desmet et al.^42^	Explored through simulation	Presence of atypical data	Simulated	Known because simulated	Dependent on simulation scenario; no specific figure given			
	Detailed information (vital signs data used as illustrative example)	Presence of atypical data	Real	Unclear (‘closer inspection’)	Reported: 83% (10/(10+2))	Reported: 99% (204/(204+2))	Calculated: 83% (10/(10+2))	Calculated: 99% (204/(204+2))
Desmet et al.^43^	Explored through simulation	Presence of atypical data	Simulated	Known because simulated	Reported: dependent on simulation scenario; no specific figure given	Reported: median specificity varied from 98%–100% depending on scenario	Not reported and not possible to calculate (results of many simulations presented)	
Knepper et al.^26^	Detailed information	Presence of fabricated data	Simulated with physician input	Known because simulated	Reported: best result from 4 scenarios (study 1): 86% (6/(6+1))	Reported: best result from 4 scenarios (study 1a): 87% (148/(148+23))^f^	Reported: best result from 4 scenarios (study 2a): 27% (3/3+8)	Reported: best result from 4 scenarios (study 1): 99% (132/132+1)
Knott et al.^48^	Partial (total number of sites not reported but likely more than number whose results reported; ‘true’ status of any unreported centres not known)	Presence of any findings	Real	On-site monitoring	Calculated: 85% (11/(11+2))	Calculated: 88% (7/(7+1))	Calculated: 92% (11/(11+1)	Calculated: 78% (7/(7+2))
		Presence of findings ‘indicative of sloppy practice’ (clearer definition not reported)	Real	On-site monitoring	Calculated: 83% (10/((10+2))	Calculated: 78% (7/(7+2))	Calculated: 83% (10/(10+2))	Calculated: 78% (7/(7+2))
		Presence of serious findings (clearer definition not reported)	Real	On-site monitoring	Calculated: 100% (1/1+0)	Calculated 45% (9/(9+11))	Calculated 8% (1/(1+11))	Calculated 100% (9/(9+0))
Lindblad et al.^50^	Partial (‘true’ status known only at 21/413 centres)	Presence of serious problems	Real	Regulatory inspection	Reported: 83% (5/((5+1))	Cannot be calculated without making assumptions about the 392/413 sites with unknown ‘true’ status		
		Presence of minor problems	Real	Regulatory inspection	Reported: 89% (8/(8+1))			
		Presence of any problems	Real	Regulatory inspection	Reported: 87% (13/(13+2))			
O’Kelly^51^	Detailed information, but sample of data from trial^g^	Presence of fabricated data	Simulated with physician input	Known because simulated	Calculated: 33% (1/(1+2))	Calculated: 95% (18/(18+1))	Calculated: 50% (1/(1+1))	Calculated: 90% (18/(18+2))
Pogue et al.^52^	POISE trial data: detailed information from all sites with >= 20 randomisations	Presence of fabricated data	Real	On-site monitoring	Reported: different models and different thresholds give different pros and cons in terms of classification. Models 1, 3 and 5 all have at least some scenarios where both specificity and sensitivity >80%. (Models 1 and 5, risk score ≥ 7; Model 3, risk score ≥ 5)			
	HOPE trial data: summary information from all sites with ≥ 20 randomisations	Presence of fabricated data	Real	On-site monitoring	N/a (no true positives)	Reported: model 1: 99% (178/(178+2))	Calculated: all models: 0% (no true positives, so any positives are false)	Calculated: all models: 100% (no true positives, so all negatives are true negatives)
Stenning et al.^16^	Partial (only sample of negative-testing sites visited, although the study design aimed to control for this)	Presence of ≥1 serious (Major or Critical) finding	Real	On-site monitoring	Calculated: primary analysis: 52% (37/(37+34))	Calculated: primary analysis: 62% (8/(8+5))	Reported: primary analysis: 88% (37/(37+5))	Calculated: primary analysis: 19% (8/(8+34))
					Calculated: secondary analysis excluding re-consent findings: 59% (36/(36+25))	Calculated: secondary analysis excluding re-consent findings: 74% (17/(17+6))	Reported: secondary analysis excluding re-consent findings: 86% (36/(36+6))	Calculated: secondary analysis excluding re-consent findings: 40% (17/(17+25))
					Calculated: secondary analysis excluding all consent findings: 60% (29/(29+19))	Calculated: secondary analysis excluding all consent findings: 64% (23/(23+13))	Reported: secondary analysis excluding all consent findings: 69% (29/(29+13))	Calculated: secondary analysis excluding all consent findings: 55% (23/(23+19))
Van den Bor et al.^58^	Partial in paper, but authors confirmed that trial implemented source data verification for all sites (personal communication)	Presence of fabricated data	Real	On-site monitoring	Various situations presented, with different implications for classification ability. Median false positives below 10% for all scenarios, lower with higher m-constant; in various situations (combinations of specific m-constants with specific scenarios), the fraudulent centre is flagged ≥ 3 times (authors’ proposed threshold) 100% of the time. Some scenarios have 100% highlighting of fraudulent centre and very low false positive rate – for example, scenario 1, m = 20, scenario 2, m = 2, scenario 3, m = 20 (all with false positive rate of 2%)			
Wu and Carlsson^60^	Partial (15/17 sites have unknown ‘true’ status)	Presence of fabricated data	Real	Auditing	Results presented narratively via a number of scenarios. For ‘angular clustering’, fourth scenario (correlation 0.7, 3 outliers) results in sensitivity, specificity, positive and negative predictive values all ≥98%. For ‘neighbourhood clustering’, specificity in all scenarios is ≥94% and second scenario (variances 0.45, 3 outliers, cluster size 27) results in sensitivity, specificity, positive and negative predictive values all ≥50%			

a Number of correctly flagged problem sites/(number of correctly flagged problem sites+sites incorrectly *not* flagged as concerning); thick border used to highlight results more than or equal to 90%.

b Number of sites correctly flagged as not concerning/(number of sites correctly flagged as not concerning+sites incorrectly flagged as concerning); thick border used to highlight results more than or equal to 90%.

c Number of correctly flagged problem sites/(number of correctly flagged problem sites+sites incorrectly flagged as concerning); thick border used to highlight results more than or equal to 90%.

d Number of sites correctly flagged as not concerning/(number of sites correctly flagged as not concerning+sites incorrectly *not* flagged as concerning); thick border used to highlight results more than or equal to 90%.

e One ‘positive’ centre is described as ‘reveal[ing that] RBM was not assessing risk sufficiently to drive monitoring decisions’.

f Publication incorrectly rounds this to 86%.

g Approximately one-third of sites included from a trial; also some uncertainty about total number of sites (sometimes reported as 21, sometimes 22; used 22 for calculations given here as this is the figure in the ‘Results’ section).

Of the eight reports with some available statistics, 1/7 had sensitivity ≥90% in at least one scenario (statistic unavailable in one report), 4/7 had specificity ≥90% in at least one scenario (unavailable in one report), 1/6 had positive predictive value ≥90% in at least one scenario (unavailable in two reports) and 5/6 had negative predictive value ≥90% in at least one scenario (unavailable in two reports). Four reports contained at least one scenario where more than one of these statistics was ≥90%, and in one case all four statistics were over 80%.^42^ All four of these reports had limitations in terms of either lack of clarity around how the ‘true’ site status was ascertained,^42^ unclear outcome measure definition,^48^ or low or unavailable results for the other classification statistics.^51,52^ The four reports all described central statistical monitoring methods (as opposed to triggered monitoring), and had used a variety of statistical techniques, including both ‘supervised’ and ‘unsupervised’ analyses.^63^

Some papers reported on actual or theoretical cost savings from reduced on-site monitoring,^36,41,44^ and others commented on the risk of incurring costs if their proposed central monitoring method identifies sites that do not in fact have meaningful problems (i.e. false positives).^26,58^ However, no papers gave detailed costings for the development, implementation and maintenance of the central monitoring methods themselves.

## Discussion

We conducted a scoping review to identify and characterise published methods for assessing the risk of not taking corrective action at trial sites at a given time. Although our search looked for reports from any time after 1995, over half of our results are from after 2013, highlighting the recent growth of risk-based monitoring concepts. Where information on host trials was available, they were almost always trials of investigational medicinal products, emphasising the interest in applying risk-based methods – and accessing the potential associated efficiency benefits – in this setting. Around a third of our results were not full, peer-reviewed reports, reflecting a wider problem with availability of evidence supporting trial conduct methods.^64^

Identified methods were mainly in two broad categories. Most were about central statistical monitoring, which uses statistical testing of all or a subset of trial data items to compare sites and identify atypical trial centres. A minority described triggered monitoring techniques, whereby sites are assessed against pre-specified site metric threshold rules (usually binary), with sites meeting the greatest number of ‘triggers’ being considered the most concerning. Several authors note that central statistical monitoring needs sufficient overall and per-site sample sizes for adequate statistical power^24,26,58^ (although some described methods were shown to detect problem sites during interim analysis or other early timepoints).^26,50,58^ Triggered monitoring, however, can be used at any stage of a trial’s recruitment (especially with trigger rules based on single instances of a given protocol violation, for example). We therefore suggest that the two techniques can, at least in theory, be used in combination.

In line with our review’s aims, our focus in characterising our results was on looking for evidence supporting the use of each proposed monitoring method. It was therefore beyond our scope to compare and contrast the different central statistical monitoring methods proposed in these reports. Several previous papers have reviewed these methods in more detail.^24,52,63,65,66^

Nearly half of the central statistical monitoring reports had a stated focus on identification of fraud or data fabrication. The possibility of fraud is a serious concern to trialists and a threat to wider trust in science.^67^ It was possibly an important factor in establishing 100% on-site verification of trial data as a common monitoring approach.^68,69^ This may help explain the prevalence of reports about fraud detection, as some may see the priority in risk-based monitoring to be establishing its fraud detection ability compared with 100% SDV. However, although the incidence of data fraud is difficult to quantify, cases of extensive data fabrication appear rare enough to have individual notoriety.^70^ Furthermore, methods to detect fraud are necessarily rather selective, and therefore may not alone be suitable for trialists looking to detect more common, lower level data integrity issues such as poor equipment calibration or inadequately trained trial staff, which central statistical monitoring methods may also be well-suited to detect.

We collected data on how the proposed methods we identified had been evaluated. A number of reports only presented proposed, untested methods, or only selected case studies to demonstrate the methods’ performance. Of those that presented more detailed evaluation, a common limitation was that the ‘true’ status both of identified problem sites and sites apparently not of concern was often not available, or only partially available. It was therefore difficult to know if the ‘concern’ status of sites in central monitoring results represented meaningful problems or not. In addition, a number of studies use simulation to create ‘true’ sites of concern; these raise the additional question of whether these simulations reflect real-life issues, though the involvement of clinicians (i.e. those who would provide real-life trial data) in the simulation process of some reports^26,51^ is reassuring.

Of the few reports with available classification statistics, the best results were often in methods’ specificity or negative predictive value. The latter finding in particular could be encouraging for those with concerns that if risk-based monitoring means reduced or omitted monitoring activity, it might fail to detect serious errors. Some of the methods also showed good classification ability in more than one classification statistic. However, this was not without caveats of opaque reporting, other classification statistics being poor or unavailable, or the potential limitations of simulation mentioned above.

It is important to recognise the limitations of the available ‘gold-standards’ in the classification of sites. When methods are tested using simulated or real-but-adjusted data, it may be difficult to know how well these accurately recreate real-life situations. When central monitoring methods are tested in real, ongoing trials, on-site monitoring may be an imperfect reference test, in that it may not be able to identify all problems. By contrast, it is clear that central monitoring, with its enhanced inter- and intra-site review, can identify issues that a single team at one site for a limited time might not.^66^

It could be argued that at least some of the methods we have identified do not need extensive evaluation because they prove their own worth. For instance, they help identify outliers that in some cases are self-evidently meaningful problems to resolve. We acknowledge that some central monitoring activities identify ‘known’ problems (e.g. identifying weekend visit dates, which are unlikely to be correct) and are valuable for data cleaning purposes. However, we were specifically interested in the more nuanced use of these methods to identify sites of ‘concern’, at which monitoring activity may be targeted, and consequently sites ‘not of concern’, monitoring of which may be reduced or omitted. In light of the limitations we have described here, we do not believe any methods have yet demonstrated sufficiently reliable classification ability to justify more widespread adoption.

Aside from some comments on the potential cost of investigating false positive central monitoring results,^26,58^ the reports we identified contained limited information on the cost of developing and implementing their methods. As well as uncertainty about how to develop relevant methods, uncertainty or concern about costs involved is a substantial barrier to adoption of risk-based monitoring.^71^

Further work is needed to fully demonstrate the effectiveness of these dynamic site risk assessment methods which, alongside pre-trial risk assessments, form the core of risk-based monitoring. We therefore recommend the following:

1. Coordinate research efforts. From the scoping review and contact with report authors, it was clear that various small research projects relevant to this topic were ongoing, but mostly in isolation. Researchers in this area should take stock of existing research, and set clear priorities to ensure research time is well-spent.
2. Standardise monitoring studies. Core outcome sets^72^ or other mechanisms to standardise studies about monitoring would improve study quality and may facilitate cross-study evidence synthesis.
3. Share evidence. Time, commercial sensitivity and perceived reputational risk could all be barriers to publishing evidence about monitoring practices. However, additional, publicly available evidence to support the best monitoring practice will allow trialists in all settings to adopt new methods with confidence.
4. Publish full papers. Conference abstracts and posters can disseminate basic information about new ideas, but rarely have enough detail to allow replication or robustly demonstrate effectiveness. As this emerging field cannot be built on abstracts alone, we encourage researchers to publish full, peer-reviewed papers about their monitoring methods.
5. Combine complementary methods. Although work has been done on a number of distinct risk-based monitoring methods, an optimal monitoring plan might involve a combination of these, including both central statistical monitoring and triggered monitoring. A collaborative approach to combining existing methods could help develop and test such an idea.

We acknowledge several limitations. Our database searches identified relevant material from disparate locations, including abstracts in conferences in unrelated research fields. It is possible that other abstract collections include relevant material, but it was not feasible to find all of these. Although the Internet searches made little contribution to the final list of included reports, they may have been limited by known reproducibility problems.^73^

Scoping review methodology advises that relevant experts in a field are surveyed to help identify other relevant work.^74^ We have not formally done this. We have, however, contacted most authors of included reports for clarifications, and this has not highlighted any additional relevant reports.

Some search results were of borderline relevance to our aims, and took discussion to ultimately include or exclude. It is possible that other researchers repeating the same review might result in a slightly different list, but we believe this might only affect the ‘method-only’ papers, which are not critical to our conclusions. The comprehensive nature of our search strategy gives us confidence that our report is a sound overview of the state of the evidence in this research area.

We have not performed a formal quality assessment of reports we found; however, this is considered by some to be unnecessary in scoping review methodology.^29^ There is also no validated way to review the quality of risk-based monitoring studies, although we used the QUADAS-2 tool to inform our data collection template.

Finally, we acknowledge that some time has passed since we first conducted our search for relevant evidence. Conscious of this, we repeated the main database search in 2018 (albeit with only one author conducting title and abstract screening) and added three relevant reports. We are not aware of any research published since then that might change our overall conclusions. If evidence is now available that addresses the limitations we have highlighted in the existing literature, we would certainly consider this a positive development.

Our scoping review highlighted some promising evidence for risk-based monitoring in ongoing trials. However, currently published methods may not yet have demonstrated their efficacy or cost-effectiveness well enough for trialists to implement them with confidence as a means to target or omit on-site visits. A more coordinated, collaborative and transparent approach to developing and sharing evidence in this field, including industry and academic partners, could help it grow beyond its current nascent state, and could contribute to risk-based monitoring more quickly entering routine practice.

## Supplemental Material

sj-pdf-1-ctj-10.1177_1740774520976561 – Supplemental material for Dynamic methods for ongoing assessment of site-level risk in risk-based monitoring of clinical trials: A scoping review Click here for additional data file. Supplemental material, sj-pdf-1-ctj-10.1177_1740774520976561 for Dynamic methods for ongoing assessment of site-level risk in risk-based monitoring of clinical trials: A scoping review by William J Cragg, Caroline Hurley, Victoria Yorke-Edwards and Sally P Stenning in Clinical Trials

sj-pdf-2-ctj-10.1177_1740774520976561 – Supplemental material for Dynamic methods for ongoing assessment of site-level risk in risk-based monitoring of clinical trials: A scoping review Click here for additional data file. Supplemental material, sj-pdf-2-ctj-10.1177_1740774520976561 for Dynamic methods for ongoing assessment of site-level risk in risk-based monitoring of clinical trials: A scoping review by William J Cragg, Caroline Hurley, Victoria Yorke-Edwards and Sally P Stenning in Clinical Trials

## References

[1] Macefield RC Beswick AD Blazeby JM, et al. A systematic review of on-site monitoring methods for health-care randomised controlled trials. Clin Trials 2013; 10(1): 104–124. 23345308 10.1177/1740774512467405

[2] Sheetz N Wilson B Benedict J, et al. Evaluating source data verification as a quality control measure in clinical trials. Ther Innov Regul Sci 2014; 48(6): 671–680. 30227471 10.1177/2168479014554400

[3] Reith C Landray M Devereaux PJ, et al. Randomized clinical trials – removing unnecessary obstacles. N Engl J Med 2013; 369: 1061–1065. 24024845 10.1056/NEJMsb1300760

[4] Duley L Antman K Arena J, et al. Specific barriers to the conduct of randomized trials. Clin Trials 2008; 5(1): 40–48. 18283079 10.1177/1740774507087704

[5] Olsen R Bihlet AR Kalakou F, et al. The impact of clinical trial monitoring approaches on data integrity and cost – a review of current literature. Eur J Clin Pharmacol 2016; 72(4): 399–412. 26729259 10.1007/s00228-015-2004-y

[6] Tudur Smith C Stocken DD Dunn J, et al. The value of source data verification in a cancer clinical trial. PLoS ONE 2012; 7(12): e51623. 23251597 10.1371/journal.pone.0051623 PMC3520949

[7] Buyse M Squifflet P Coart E, et al. The impact of data errors on the outcome of randomized clinical trials. Clin Trials 2017; 14(5): 499–506. 28641461 10.1177/1740774517716158

[8] MRC/DH/MHRA Joint Project. Risk-adapted Approaches to the Management of Clinical Trials of Investigational Medicinal Products, 2011, https://www.gov.Uk/government/uploads/system/uploads/attachment_data/file/343677/Risk-adapted_approaches_to_the_management_of_clinical_trials_of_investigational_medicinal_products.pdf (accessed 8 September 2017).

[9] Food and Drug Administration. Guidance for industry: oversight of clinical investigations – a risk-based approach to monitoring, 2013, https://www.fda.gov/media/116754/download (accessed 8 September 2017).

[10] European Medicines Agency. Reflection paper on risk based quality management in clinical trials, 2013, http://www.ema.europa.Eu/docs/en_GB/document_library/Scientific_guideline/2013/11/WC500155491.pdf (accessed 16 January 2017).

[11] Brosteanu O Houben P Ihrig K, et al. Risk analysis and risk adapted on-site monitoring in noncommercial clinical trials. Clin Trials 2009; 6(6): 585–596. 19897532 10.1177/1740774509347398

[12] McMahon AD Conway DI Macdonald TM, et al. The unintended consequences of clinical trials regulations. PLoS Med 2009; 6(11): e1000131. 10.1371/journal.pmed.1000131 PMC2768793 19918557

[13] Salman RA-S Beller E Kagan J, et al. Increasing value and reducing waste in biomedical research regulation and management. Lancet 2014; 383: 176–185. 24411646 10.1016/S0140-6736(13)62297-7 PMC3952153

[14] Brosteanu O Schwarz G Houben P, et al. Risk-adapted monitoring is not inferior to extensive on-site monitoring: results of the ADAMON cluster-randomised study. Clin Trials 2017; 14: 584–596. 28786330 10.1177/1740774517724165 PMC5718334

[15] Journot V Pignon JP Gaultier C, et al. Validation of a risk-assessment scale and a risk-adapted monitoring plan for academic clinical research studies – the Pre-Optimon study. Contemp Clin Trials 2011; 32: 16–24. 20951234 10.1016/j.cct.2010.10.001

[16] Stenning SP Cragg WJ Joffe N, et al. Triggered or routine site monitoring visits for randomised controlled trials: results of TEMPER, a prospective, matched-pair study. Clin Trials 2018; 15: 600–609. 30132361 10.1177/1740774518793379 PMC6236642

[17] Sullivan LB. The current status of risk-based monitoring, http://www.appliedclinicaltrialsonline.com/current-status-risk-based-monitoring (accessed 6 June 2018).

[18] Mealer M Kittelson J Thompson BT, et al. Remote source document verification in two national clinical trials networks: a pilot study. PLoS ONE 2013; 8(12): e81890. 24349149 10.1371/journal.pone.0081890 PMC3857788

[19] Uren SC Kirkman MB Dalton BS, et al. Reducing clinical trial monitoring resource allocation and costs through remote access to electronic medical records. J Oncol Pract 2013; 9(1): e13–e16. 23633977 10.1200/JOP.2012.000666 PMC3545670

[20] Weston W Smedley J Bennett A, et al. The Cooking and Pneumonia Study (CAPS) in Malawi: implementation of remote source data verification. PLoS ONE 2016; 11(6): e0155966. 27355447 10.1371/journal.pone.0155966 PMC4927187

[21] MHRA. Good Clinical Practice guide. London: Stationery Office, 2012.

[22] Lad PM Dahl R. Audit of the informed consent process as a part of a clinical research quality assurance program. Sci Eng Ethics 2014; 20(2): 469–479. 23975172 10.1007/s11948-013-9461-4

[23] International Conference on Harmonisation of Technical Requirements for Pharmaceuticals for Human Use (ICH). Integrated Addendum to ICH E6(R1): guideline for good clinical practice E6(R2), 2016, https://database.ich.org/sites/default/files/E6_R2_Addendum.pdf (accessed 25 February 2017).

[24] Kirkwood AA Cox T Hackshaw A. Application of methods for central statistical monitoring in clinical trials. Clin Trials 2013; 10(5): 783–806. 24130202 10.1177/1740774513494504

[25] Tudur Smith C Williamson P Jones A, et al. Risk-proportionate clinical trial monitoring: an example approach from a non-commercial trials unit. Trials 2014; 15: 1–10. 24739398 10.1186/1745-6215-15-127 PMC4022377

[26] Knepper D Lindblad AS Sharma G, et al. Statistical monitoring in clinical trials: best practices for detecting data anomalies suggestive of fabrication or misconduct. Ther Innov Regul Sci 2016; 50: 144–154. 30227005 10.1177/2168479016630576

[27] Hurley C Shiely F Power J, et al. Risk based monitoring (RBM) tools for clinical trials: a systematic review. Contemp Clin Trials 2016; 51: 15–27. 27641969 10.1016/j.cct.2016.09.003

[28] Daudt HM van Mossel C Scott SJ. Enhancing the scoping study methodology: a large, inter-professional team’s experience with Arksey and O’Malley’s framework. BMC Med Res Methodol 2013; 13: 48. 23522333 10.1186/1471-2288-13-48 PMC3614526

[29] Pham MT Rajić A Greig JD, et al. A scoping review of scoping reviews: advancing the approach and enhancing the consistency. Res Synth Methods 2014; 5(4): 371–385. 26052958 10.1002/jrsm.1123 PMC4491356

[30] International Conference on Harmonisation of Technical Requirements for Pharmaceuticals for Human Use (ICH). Guideline for Good Clinical Practice E6(R1), 1996, https://www.ema.europa.eu/en/documents/scientific-guideline/ich-e6-r1-guideline-good-clinical-practice_en.pdf (accessed 8 September 2017).

[31] Devito Dabbs A Song MK Hawkins R, et al. An intervention fidelity framework for technology-based behavioral interventions. Nurs Res 2011; 60(5): 340–347. 21878796 10.1097/NNR.0b013e31822cc87d PMC3164967

[32] Opler MGA Yavorsky C Daniel DG. Positive and Negative Syndrome Scale (PANSS) training: challenges, solutions, and future directions. Innov Clin Neurosci 2017; 14: 77–81. http://www.ncbi.nlm.nih.gov/pubmed/29410941 (accessed 4 Jun 2018). 29410941 PMC5788255

[33] Bakobaki J Joffe N Burdett S, et al. A systematic search for reports of site monitoring technique comparisons in clinical trials. Clin Trials 2012; 9(6): 777–780. 23059772 10.1177/1740774512458993

[34] Whiting PF Rutjes AWS Westwood ME, et al. QUADAS-2: a revised tool for the quality assessment of diagnostic accuracy studies. Ann Intern Med 2011; 155: 529–536. 22007046 10.7326/0003-4819-155-8-201110180-00009

[35] Moher D Liberati A Tetzlaff J, et al. Preferred reporting items for systematic reviews and meta-analyses: the PRISMA statement. PLoS Med 2009; 6: e1000097. 19621072 10.1371/journal.pmed.1000097 PMC2707599

[36] Agrafiotis DK Lobanov VS Farnum MA, et al. Risk-based monitoring of clinical trials: an integrative approach. Clin Ther 2018; 40(7): 1204–1212. 30100201 10.1016/j.clinthera.2018.04.020

[37] Almukhtar T Glassman A. Monitoring of adverse events reporting in multicenter clinical trials using a mixed effect regression model. In: Proceedings of the 36th annual meeting of the Society for Clinical Trials, Arlington, VA, 17–20 5 2015, p. 45. Arlington Heights, IL: Society for Clinical Trials.

[38] Atanu B Savanur S Suman K. Risk based monitoring in clinical trial: an application with neural networking. Biostat Biom 2017; 3: 555624.

[39] Bailey L Brudenell Straw FK George SE. Implementing a risk based monitoring approach in the early phase myeloma portfolio at Leeds CTRU. Trials 2017; 18(Suppl. 1): 220.28514964

[40] Bengtsson SKS. Risk based monitoring in clinical studies – improving data quality, 2017, https://lup.lub.lu.se/student-papers/search/publication/8928535

[41] Biglan KM Brocht A Raca P, et al. Implementing risk-based monitoring (RBM) in STEADY-PD III, a phase III multi-site clinical drug trial for Parkinson disease. Mov Disord 2016; 31: E10.

[42] Desmet L Venet D Doffagne E, et al. Linear mixed-effects models for central statistical monitoring of multicenter clinical trials. Stat Med 2014; 33: 5265–5279. 25213096 10.1002/sim.6294

[43] Desmet L Venet D Doffagne E, et al. Use of the beta-binomial model for central statistical monitoring of multicenter clinical trials. Stat Biopharm Res 2017; 9: 1–11. 10.1002/sim.6294 25213096

[44] Diani CA Rock A Moll P. An evaluation of the effectiveness of a risk-based monitoring approach implemented with clinical trials involving implantable cardiac medical devices. Clin Trials 2017; 14(6): 575–583. 28819983 10.1177/1740774517723589

[45] Djali S Janssens S Van Yper S, et al. How a data-driven quality management system can manage compliance risk in clinical trials. Drug Inf J 2010; 44: 359–373.

[46] Dress J Harnischmacher U Weiss C, et al. High quality risk management for clinical trials: use the data at your hands to manage risk in your clinical trials. Clin Trials 2011; 8: 469.

[47] Edwards P Shakur H Barnetson L, et al. Central and statistical data monitoring in the Clinical Randomisation of an Antifibrinolytic in Significant Haemorrhage (CRASH-2) trial. Clin Trials 2013; 11: 336–343. 10.1177/1740774513514145 24346610

[48] Knott C Valdes-Marquez E Landray M, et al. Improving efficiency of on-site monitoring in multicentre clinical trials by targeting visits. Trials 2015; 16: 1.25971836

[49] Kodama A Cavalcanti A Buehler A, et al. Application of simple statistical methods to evaluated possible data fabrication: an example using the act trial. Clin Trials 2010; 7: 445.

[50] Lindblad AS Manukyan Z Purohit-Sheth T, et al. Central site monitoring: results from a test of accuracy in identifying trials and sites failing Food and Drug Administration inspection. Clin Trials 2014; 11(2): 205–217. 24296321 10.1177/1740774513508028

[51] O’Kelly M. Using statistical techniques to detect fraud: a test case. Pharm Stat 2004; 3: 237–246.

[52] Pogue JM Devereaux P Thorlund K, et al. Central statistical monitoring: detecting fraud in clinical trials. Clin Trials 2013; 10(2): 225–235. 23283577 10.1177/1740774512469312

[53] Smith A Seltzer J. An in-process scaling model: a potential framework for data monitoring committees and clinical trial quality improvement. Drug Inf J 2012; 46: 8–12.

[54] Taylor RN McEntegart DJ Stillman EC, et al. Statistical techniques to detect fraud and other data irregularities in clinical questionnaire data. Drug Inf J 2002; 36: 115–125.

[55] Timmermans C Doffagne E Venet D, et al. Statistical monitoring of data quality and consistency in the Stomach Cancer Adjuvant Multi-institutional Trial Group Trial. Gastric Cancer 2016; 19(1): 24–30. 26298185 10.1007/s10120-015-0533-9

[56] Valdes-Marquez E Hopewell JC Landry M, et al. A key risk indicator approach to central statistical monitoring in multicentre clinical trials: method development in the context of an ongoing large-scale randomized trial. Trials 2011; 12: A135.

[57] Valdes-Marquez E Hopewell JC Landry M. Central statistical monitoring in multicentre clinical trials: developing statistical approaches for analysing key risk indicators. Trials 2013; 14: 267.23965227

[58] Van den Bor RM Vaessen PWJ Oosterman BJ, et al. A computationally simple central monitoring procedure, effectively applied to empirical trial data with known fraud. J Clin Epidemiol 2017; 87: 59–69. 28412468 10.1016/j.jclinepi.2017.03.018

[59] Whitham D Turzanski J Bradshaw L, et al. Development of a standardised set of metrics for monitoring site performance in multicentre randomised trials: a Delphi study. Trials 2018; 19: 557. 30326967 10.1186/s13063-018-2940-9 PMC6192223

[60] Wu X Carlsson M. Detecting data fabrication in clinical trials from cluster analysis perspective. Pharm Stat 2011; 10(3): 257–264. 20936626 10.1002/pst.462

[61] Zink RC Dmitrienko A Dmitrienko A. Rethinking the clinically based thresholds of TransCelerate BioPharma for risk-based monitoring. Ther Innov Regul Sci 2018; 52(5): 560–571. 29714565 10.1177/2168479017738981

[62] Organisation for Economic Co-operation and Development. OECD recommendation on the governance of clinical trials, 2013, https://www.oecd.org/sti/sci-tech/oecd-recommendation-governance-of-clinical-trials.pdf

[63] Oba K. Statistical challenges for central monitoring in clinical trials: a review. Int J Clin Oncol 2016; 21(1): 28–37. 26499195 10.1007/s10147-015-0914-4

[64] Brueton V Tierney JF Stenning S, et al. Identifying additional studies for a systematic review of retention strategies in randomised controlled trials: making contact with trials units and trial methodologists. Syst Rev 2017; 6: 167. 28830570 10.1186/s13643-017-0549-9 PMC5568351

[65] Timmermans C Venet D Burzykowski T. Data-driven risk identification in phase III clinical trials using central statistical monitoring. Int J Clin Oncol 2016; 21(1): 38–45. 26233672 10.1007/s10147-015-0877-5

[66] Venet D Doffagne E Burzykowski T, et al. A statistical approach to central monitoring of data quality in clinical trials. Clin Trials 2012; 9(6): 705–713. 22684241 10.1177/1740774512447898

[67] Buyse M Evans SJW Buyse M, et al. Fraud in clinical trials. In: Armitage P Colton T (eds) Encyclopedia of biostatistics. Chichester: John Wiley & Sons, Ltd, 2005, pp. 2023–2031.

[68] Peto R Collins R Sackett D, et al. The trials of Dr. Bernard Fisher: a European perspective on an American episode. Control Clin Trials 1997; 18(1): 1–13. 9055048 10.1016/s0197-2456(96)00225-5

[69] Seachrist L. Scientific misconduct. NIH tightens clinical trials monitoring. Science 1994; 264: 499–500. 10.1126/science.8160006 8160006

[70] George SL Buyse M. Data fraud in clinical trials. Clin Invest 2015; 5: 161–173. 10.4155/cli.14.116 PMC4340084 25729561

[71] Hurley C Sinnott C Clarke M, et al. Perceived barriers and facilitators to risk based monitoring in academic-led clinical trials: a mixed methods study. Trials 2017; 18: 423. 28893317 10.1186/s13063-017-2148-4 PMC5594426

[72] Williamson PR Altman DG Blazeby JM, et al. Developing core outcome sets for clinical trials: issues to consider. Trials 2012; 13: 132. 22867278 10.1186/1745-6215-13-132 PMC3472231

[73] Ćurković M Košec A. Bubble effect: including internet search engines in systematic reviews introduces selection bias and impedes scientific reproducibility. BMC Med Res Methodol 2018; 18: 130. 30424741 10.1186/s12874-018-0599-2 PMC6234590

[74] Arksey H O’Malley L. Scoping studies: towards a methodological framework. Int J Soc Res Methodol 2005; 8: 19–32.

