# Supplementary material for: Dynamic methods for ongoing assessment of site-level risk in risk-based monitoring of clinical trials: A scoping review
Source: Clin Trials. 2021 Feb 20;18(2):245–59. doi: 10.1177/1740774520976561 (PMC8010889; doi:10.1177/1740774520976561)
Supplement: sj-pdf-1-ctj-10.1177_1740774520976561 – Supplemental material for Dynamic methods for ongoing assessment of site-level risk in risk-based monitoring of clinical trials: A scoping review [file sj-pdf-1-ctj-10.1177_1740774520976561.pdf]

Annotated list of data collection variables (highlighted fields have been included in final report)

Field	Format	Comments
**1 Report characteristics:** to gather basic information about the reports and the trials that described methods had been used in		
Record Number	Integer	
Title	Free text	Article title
First author	Free text	
Year	Numerical	Year of article publication
Journal	Free text	
Type of publication	Category:<br>- Conference abstract/poster<br>- Peer-reviewed paper<br>- Thesis<br>- Other	
Setting/disease (if any)	Free text	Setting/disease area of trials that the monitoring method(s) had been used in
Geographical setting/country	Free text	Geographical setting/country of the trials that the monitoring method(s) had been used in
IMP/non-IMP	Category:<br>- IMP<br>- No IMP<br>- Not known or no specific trial involved	Whether or not the trials that the monitoring method(s) had been used in involved Investigational Medicinal Product(s)
Phase of trial	Category:<br>- I<br>- II<br>- III<br>- Not known or no specific trial involved	Phase of the trials that the monitoring method(s) had been used in; categories not mutually exclusive
Risk category/status of intervention	Category:<br>- A (licensed IMP, used within its licensed indication)<br>- B (licensed IMP, used outside its licensed indication)<br>- C (unlicensed IMP)<br>- Not known	Intervention risk category of the trials that the monitoring method(s) had been used in, according to the Organisation for Economic Co-operation and Development*; categories not mutually exclusive<br><br>* https://www.oecd.org/sti/sci-tech/oecd-recommendation-governance-of-clinical-trials.pdf

Field	Format	Comments
**2 Detail of reports' focus and scope, and any assessment of methods' effectiveness:** to gather information on the type of method described, on whether it was descriptive only or also included some assessment of how well the method works and, if some assessment done, what form the assessment took. The category questions describing the type of assessment were not mutually exclusive.		
Focus of work	Category:<br>- Central statistical monitoring, with focus on fraud or misconduct<br>- Central statistical monitoring, general<br>- Triggered monitoring<br>- Other flagging/targeting method<br>- Other	"Central statistical monitoring": methods involving statistical testing to identify outlying or unusual clinical trial centres. Reports about fraud or data fabrication differentiated because they were assumed to use different methods and different thresholds for defining 'problem centres' compared to methods looking for any type of problem.<br><br>"Triggered monitoring": use of threshold-based rules to identify problem centres (e.g. those with data return <80% or an unusually high number of serious adverse events submitted might be flagged).
If other, explain	Free text	
Scope of work	Category:<br>- Theory only<br>- Association between central monitoring finding and site feature<br>- Description/development of method<br>- Some assessment of effectiveness	"Theory only": papers discussing potential risk-based monitoring methods without any concrete evidence generation.<br><br>"Association between central monitoring finding and site feature": as a hypothetical example, papers linking high or low recruitment with the number of protocol violations, without then developing any monitoring method based on this.<br><br>"Theory only" and "Association…" papers were ultimately excluded from final results
If some assessment of effectiveness, case studies presented?	Category:<br>- Yes<br>- No	Case studies defined as selected instances illustrating (usually narratively) how a method works.
If some assessment of effectiveness, method explored on real data with no known fraud or other serious problems?	Category:<br>- Yes<br>- No	Method tried out on real trial data without any known problems, i.e. no 'true positive' problem centres to find. Any 'positive' centres flagged through the central monitoring method might be assumed to be false positives without further investigation.

Field	Format	Comments
If some assessment of effectiveness, method used to find simulated fabrication/fraud?	Category:<br>- Yes<br>- No	Real datasets modified to simulate fabricated data, then attempts made to identify the fabricated data using the monitoring method.
If some assessment of effectiveness, method used to find known problems in real data?	Category:<br>- Yes<br>- No	Datasets obtained from trials with known instances of fraud, data fabrication or other issues; method used to identify the problem centres (whether or not done by individuals blind to which the problem centres were).
If some assessment of effectiveness, method implemented in a trial and results of targeted on-site monitoring reported?	Category:<br>- Yes<br>- No	Results of on-site monitoring reported, i.e. number of (serious) findings from visits.
If some assessment of effectiveness, method implemented in a trial and effects on trial reported, in terms of cost, data quality or something else?	Category:<br>- Yes<br>- No	Effects of monitoring method on the trial, usually suggesting that risk-based monitoring methods reduce costs, improve aspects of trial quality, or both.
If some assessment of effectiveness, prospectively designed, controlled study to look at predictive ability of targeted on-site monitoring methods?	Category:<br>- Yes<br>- No	Use of method in a prospectively designed experiment aiming to assess how well it correctly identifies problem sites and excludes non-problem sites
**3 Quality assessment:** these fields were developed following review of the QUADAS-2 tool for quality assessment of diagnostic accuracy studies* because we suggest that sort of study shares similar potential sources of bias as the sort of study we were looking for. As it was not within the scope of our project to validate these questions as a quality assessment tool in this setting, we have not ultimately reported this information. However, it has informed our interpretation of the limitations in the existing evidence base.<br><br>*Whiting PF, Rutjes AWS, Westwood ME, *et al.* QUADAS-2: A Revised Tool for the Quality Assessment of Diagnostic Accuracy Studies. *Ann Intern Med* 2011;**155**:529. doi:10.7326/0003-4819-155-8-201110180-00009		
Comments on the quality / limitations of the evidence presented	Free text	General comments on quality
If simulated data used, are simulation methods well-described and well-justified? Is there any risk of introducing bias?	Free text	Example of potential problem: simulated data used, but no attempt to make it reflect a possible real-life situation (e.g. extreme outliers added to data, when deliberate fabrication might involve addition of ‘normal’-looking data)

Field	Format	Comments
If simulated data used, were outcome assessors blinded to simulation methods and details of any sites with implanted fabrication?	Free text	Example of potential problem: if outcome assessors – those using the proposed central monitoring method to identify problem centres – knew the simulation method, or knew the number of centres they were looking for, this might make it easier for them to guess which the problem centres were.
If tested method using dataset with known fraud or other issues, is the choice of 'reference test' (usually source data verification) well-described and well-justified?	Free text	Example of potential problem: real fraud, data fabrication or other issues might be expected to have been found through on-site monitoring activities. A potential problem might be that this is not clearly described in a paper, so it is not possible to confirm how we know the 'true' status of each centre.
If tested method using dataset with known fraud or other issues, are the results of the method being evaluated being assessed without knowledge of 'reference test' results? E.g. are statisticians trying to identify problem sites blinded to which were actually problematic?	Free text	Example of potential problem: if outcome assessors using the proposed central monitoring method to identify problem centres are not blinded to which are the problem centres, it is harder to say that the method alone has identified the problem centres.
For tests of triggered monitoring only, are thresholds for site triggering affected by subjective judgements?	Free text	Example of potential problem: if choices about which centres to target for on-site monitoring are affected by subjective assessments, this may be biased (and perhaps harder to replicate in a different setting).
For tests of triggered monitoring only, is there a fixed definition of 'negative' or 'control' sites, or is it moveable or subjective or unclear?	Free text	Example of potential problem: 'control' sites are poorly defined, harming replicability and making it difficult to assess their suitability as experimental controls.
For tests of triggered monitoring only, are people collecting outcome data blinded to site categorisation? (e.g. do they know if a visit is triggered or control?)	Free text	Example of potential problem: people collecting outcome data (for example, on-site monitors) are aware of site categorisation and this affects how they carry out the site visits, either knowingly or unknowingly.
For tests of triggered monitoring only, are people assessing outcome data (if different) blinded to site categorisation?	Free text	Example of potential problem: people assessing the outcome data (for example, oversight committees) are aware of site categorisation and this biases their outcome assessments.

Field	Format	Comments
For tests of triggered monitoring only, are site staff blinded to site categorisation?	Free text	Example of potential problem: if site staff know they are at a 'problem' centre (or a non-problem centre), they may behave differently during an on-site visit, which could affect the results of the visit.
For tests of triggered monitoring only, are outcome assessors and outcome data collectors (if different) aware of outcomes at other control sites or triggered sites?	Free text	Example of potential problem: outcome data collectors are aware of accumulating data from other site visits, and perception of a trend towards a difference (or no difference) between triggered and untriggered visits influences how subsequent visits are conducted.
For any study with defined outcome measure (i.e. not including anything explorative), how subjective is the outcome measure?	Free text	Example of potential problem: subjective outcome measures are at higher risk of bias and would need additional controls to guard against this.
Based on these quality assessment fields, what is the overall quality of the study?	Free text	Summary of quality issues
**4 Other fields:** to gather various other pieces of information about the reports. Some of these have not ultimately been reported (but data are available on reasonable request). The fields on classification ability were key to our study, however.		
Give aims/objectives, or if none, last sentence(s) of intro where they explain scope of paper	Free text	This was gathered to inform our understanding of the scope of each report. Not ultimately reported.
Summary of aims/objectives?	Free text	Summary and interpretation of the field above. Not ultimately reported.
Description of monitoring method proposed (including triggers/central statistical monitoring elements used)	Free text	A brief summary of the methods proposed. Not ultimately reported, partly because previously published papers have already included useful reviews of central statistical monitoring methods.
"Triggers" mentioned, if any?	Free text	Details of any mentioned triggers (threshold-based rules for distinguishing problem sites from well-performing sites). Not ultimately reported.
For central statistical monitoring papers, broadly what issues did they look at?	Free text	For example, inliers and outliers, digit preference and so on. Not ultimately reported, partly because previously published papers have already included useful reviews of central statistical monitoring methods.

Field	Format	Comments
Describe as 'supervised' or 'unsupervised' analyses?	Category:<br>- Supervised<br>- Unsupervised<br>- Both<br>- Unclear	As defined in previously-published work*, 'unsupervised' analyses involves looking through all trial data for unusual patterns; in 'supervised' analyses, by contrast, analysts build in pre-specified limits to what is included (e.g. limits on how much data is included in the analysis, or pre-specified risk thresholds regardless of sample size).<br><br>We did not ultimately report this because a) it was not always straightforward to say whether a method was supervised or unsupervised, especially given slightly different definitions in the literature, and b) although we considered the distinction to be of some interest, we agreed it was just a way of characterising the methods we identified, rather than a key finding in our work.<br><br>* Oba K. Statistical challenges for central monitoring in clinical trials: a review. *Int J Clin Oncol* 2016;**21**:28–37.
What evaluation?	Free text	This was gathered to inform our understanding of the level and nature of any evaluation of methods' effectiveness. Not ultimately reported.
What claims made about effectiveness?	Free text	We used this to collect quotes from each report about the effectiveness of the proposed methods. This informed our understanding of the scope of each work, but we have not ultimately reported this data.
Summarise predictive value info in paper?	Free text	This was gathered to inform our understanding of how much information was in each report about the ability of the methods to correctly predict the 'true' status of each site (i.e. the classification ability, as per the fields that follow). This was for discussion purposes only and has not ultimately been reported.
Category for classification info	Category:<br>- No evaluation<br>- No information on true status<br>- Partial	A category field to describe how much information was in each report about the methods' classification ability.

Field	Format	Comments
	- Explored through simulation<br>- Case studies presented only<br>- Detailed information	'True status' means whether or not each clinical trial site is confirmed to be a 'problem site' (however this is defined in each case), on the basis that central monitoring methods to flag possible problem sites are analogous to diagnostic tests.<br><br>'Partial' means information is only available on some sites (i.e. on their test results, their true status, or the total number of sites, or all of these).<br><br>'Explored through simulation' means information on statistics such as sensitivity and specificity is available for a range of simulated scenarios (though with limited or no information from real-life settings).<br><br>'Case studies presented only' means only a few, selected examples of methods' capabilities is presented.<br><br>'Detailed information' means information available to give a full (or at least detailed) picture of methods' sensitivity, specificity, and positive and negative predictive values, from specifically tested situations (as opposed to extensive simulation).
Best classification results, if possible	Free text	We gathered and have reported information from each paper on the best (i.e. most successful) classification results in each report. In some cases this is reported directly from the paper, in others we calculated it from information available in the paper. This is reported, with details of any calculations, in Table 4 of our report.
What classification terms mentioned?	Free text	We gathered information on use of terminology (e.g. presence or absence of 'sensitivity', 'specificity' etc) to inform our understanding of each report. We have not ultimately reported this.

Field	Format	Comments
Any information provided on cost/resource implications?	Free text	We gathered information on cost or resource implications from each paper. This could either be cost of developing the methods or any related computer systems, or cost implications of adopting risk-based monitoring methods, or anything else. This is briefly reported in our manuscript.
Any comparison made between the centralised method and on-site monitoring, in any outcome?	Free text	We were interested to see if any authors had directly compared targeted and untargeted monitoring methods in terms of a monitoring-based outcome, such as ability to detect serious findings, or the time between protocol violation and its detection. This did not ultimately yield useful information, so we have not reported it.
Does it meet any of the aims of Centralised Monitoring as defined in ICH GCP?<br><br>a) identify missing data, inconsistent data, data outliers, unexpected lack of variability and protocol deviations<br><br>b) examine data trends such as the range, consistency, and variability of data within and across sites<br><br>c) evaluate for systematic or significant errors in data collection and reporting at a site or across sites; or potential data manipulation or data integrity problems<br><br>d) analyse site characteristics and performance metrics<br><br>e) select sites and/or processes for targeted on-site monitoring	Category for each aim:<br>- Yes<br>- No	We decided not to report the data from these fields because the ICH GCP aims are complex and not mutually exclusive; this made it challenging to reach agreement on which applied to each report.
Any restrictions placed on how/when method could be used?	Free text	Restrictions or limitations stated by the authors of each paper, for example if method can only be used for continuous or binary data. Not ultimately reported.

Field	Format	Comments
Other comments	Free text	Any general comments. These informed interpretation of our results.
