# Supplementary material for: Dynamic methods for ongoing assessment of site-level risk in risk-based monitoring of clinical trials: A scoping review
Source: Clin Trials. 2021 Feb 20;18(2):245–59. doi: 10.1177/1740774520976561 (PMC8010889; doi:10.1177/1740774520976561)
Supplement: sj-pdf-2-ctj-10.1177_1740774520976561 – Supplemental material for Dynamic methods for ongoing assessment of site-level risk in risk-based monitoring of clinical trials: A scoping review [file sj-pdf-2-ctj-10.1177_1740774520976561.pdf]

MEDLINE via Ovid

1. **clinical trial*.ab,ti.**
2. **Clinical Trials as Topic/**
3. **trial.ti.**
4. **randomi?ed trial*.ab,ti.**
5. **randomized controlled trial/**
6. **randomi?ed controlled trial*.ab,ti.**
7. **control trial.ab,ti.**
8. **controlled clinical trial.ab,ti.**
9. **controlled clinical trial/**
10. **clinical research study.ab,ti.**
11. **1 or 2 or 3 or 4 or 5 or 6 or 7 or 8 or 9 or 10**
12. **risk based monitoring.ab,ti.**
13. **(data monitoring not committee* not board*).ab,ti.**
14. **(remote monitoring not card* not tele* not blood).ab,ti.**
15. **(statistical adj3 monitoring).ab,ti.**
16. **risk adapted monitoring.ab,ti.**
17. **site monitoring.ab,ti.**
18. **clinical trial monitoring.ab,ti.**
19. **(central* adj4 monitor*).ab,ti.**
20. **central* statis* monitor*.ab,ti.**
21. **(trigger* adj3 monitor*).ab,ti.**
22. **supervision.ab,ti.** [1]
23. **oversight.ab,ti.** [1]
24. **key risk indicator.ab,ti.**
25. **performance indicator*.ab,ti.**
26. **performance metric.ab,ti.**
27. **monitor* method*.ab,ti.** [1]
28. **monitor* strategy.ab,ti.**
29. **(target* adj3 site monitor*).ab,ti.**
30. **quality assurance.ab,ti.** [1]
31. **quality control.ab,ti.** [1]
32. **quality management.ab,ti.** [1]
33. **site performance.ab,ti.**

34. **cent* performance.ab,ti.**
35. **quality metric*.ab,ti.**
36. **(trial performance not time trial).ab,ti.**
37. **data fabrication.ab,ti.**
38. **intercent* variance.ab,ti.**
39. **data anomal*.ab,ti.**
40. **(detect* adj2 fraud).ab,ti.**
41. **error detection.ab,ti.**
42. **quality management system.ab,ti.**
43. **12 or 13 or 14 or 15 or 16 or 17 or 18 or 19 or 20 or 21 or 24 or 25 or 26 or 27 or 28 or 29 or 32 or 33 or 34 or 35 or 36 or 37 or 38 or 39 or 40 or 41 or 42** [2]
44. **11 and 43** [2]
45. **12 or 13 or 14 or 15 or 16 or 17 or 18 or 19 or 20 or 21 or 24 or 25 or 26 or 28 or 29 or 33 or 34 or 35 or 36 or 37 or 38 or 39 or 40 or 41 or 42**
46. **45 and 11**

[1] Terms omitted from final search term due to low specificity (i.e. returned many irrelevant results).

[2] Combination terms not ultimately used as too many results obtained; presented here for transparency. Final search term 46 omits some low-specificity terms.
